# Mutation of HIV-1 Genomes in a Clinical Population Treated with the Mutagenic Nucleoside KP1461

**DOI:** 10.1371/journal.pone.0015135

**Published:** 2011-01-14

**Authors:** James I. Mullins, Laura Heath, James P. Hughes, Jessica Kicha, Sheila Styrchak, Kim G. Wong, Ushnal Rao, Alexis Hansen, Kevin S. Harris, Jean-Pierre Laurent, Deyu Li, Jeffrey H. Simpson, John M. Essigmann, Lawrence A. Loeb, Jeffrey Parkins

**Affiliations:** 1 Department of Microbiology, University of Washington, School of Medicine, Seattle, Washington, United States of America; 2 Department of Medicine, University of Washington, School of Medicine, Seattle, Washington, United States of America; 3 Department of Pathology, University of Washington, School of Medicine, Seattle, Washington, United States of America; 4 Department of Biostatistics, University of Washington, School of Medicine, Seattle, Washington, United States of America; 5 Koronis Pharmaceuticals, Redmond, Washington, United States of America; 6 Department of Chemistry, Massachusetts Institute of Technology, Cambridge, Massachusetts, United States of America; 7 Department of Biological Engineering, Massachusetts Institute of Technology, Cambridge, Massachusetts, United States of America; University of Minnesota, United States of America

## Abstract

The deoxycytidine analog KP1212, and its prodrug KP1461, are prototypes of a new class of antiretroviral drugs designed to increase viral mutation rates, with the goal of eventually causing the collapse of the viral population. Here we present an extensive analysis of viral sequences from HIV-1 infected volunteers from the first “mechanism validation” phase II clinical trial of a mutagenic base analog in which individuals previously treated with antiviral drugs received 1600 mg of KP1461 twice per day for 124 days. Plasma viral loads were not reduced, and overall levels of viral mutation were not increased during this short-term study, however, the mutation spectrum of HIV was altered. A large number (N = 105 per sample) of sequences were analyzed, each derived from individual HIV-1 RNA templates, after 0, 56 and 124 days of therapy from 10 treated and 10 untreated control individuals (>7.1 million base pairs of unique viral templates were sequenced). We found that private mutations, those not found in more than one viral sequence and likely to have occurred in the most recent rounds of replication, increased in treated individuals relative to controls after 56 (p = 0.038) and 124 (p = 0.002) days of drug treatment. The spectrum of mutations observed in the treated group showed an excess of A to G and G to A mutations (p = 0.01), and to a lesser extent T to C and C to T mutations (p = 0.09), as predicted by the mechanism of action of the drug. These results validate the proposed mechanism of action in humans and should spur development of this novel antiretroviral approach.

## Introduction

Evolution of HIV-1 proceeds about 1 million times faster than that of the human genome, with approximately one error incorporated into the viral genome each time the virus is replicated [1]. By generating diversity within the viral population, the high rate of mutagenesis facilitates continuing HIV survival in the host, even when the virus faces a complex selective environment that includes: 1) limited target cell availability and susceptibility, 2) the presence of vigorous antiviral immune responses, and 3) host intervention with multiple antiretroviral therapies [2]–[5]. Thus, an intricate and balanced network of selective forces continually acts on the HIV population to maintain an adequate rate of replication in a hostile and ever-changing host environment *in vivo*. A viral quasispecies emerges within the infected host that is composed of a spectrum of mutants that may include biological variants such as immunologic escape mutants, faster and slower growing viruses, defective viruses, and drug resistant and sensitive viruses [4]–[7]. Deleterious, neutral, advantageous, and compensatory mutants can all occur within this spectrum, resulting in a population that is largely replication defective [8]. In quasispecies, the population of relatively poorly replicating viruses nonetheless provide genetic flexibility to facilitate survival within, and rapid adaptation to, a changing selective environment [9]. This flexibility is facilitated by mutational exploration of evolutionary space and is enhanced by a rate of genetic exchange between HIV strains *in vivo* that is about an order of magnitude faster than the mutation fixation rate [10], [11].

Overall, many RNA virus populations, including HIV, appear to exist near the brink of survivability [12], [13], as agents that disrupt the delicately balanced networks described above – by increasing the frequency of mutations in the HIV genome by as little as <2-fold – cause viral extinction in cell culture [14]–[17]. Similarly, small increases in viral mutation frequencies have been shown to be associated with population collapse in other viral systems, including Vesicular Stomatitis, polio, Hepatitis C, Hantaan and foot-and-mouth disease [18]–[25].

We are studying the use of first-in-class nucleotide analogs that are incorporated by reverse transcriptase without leading to chain termination, yet base pair ambiguously and thus cause mutations, with the goal of eventually pushing the viral quasispecies beyond the brink of survivability *in vivo*. We term this approach to HIV therapy as “viral decay acceleration” (VDA). Studies with 1 mM 5-hydroxydeoxycytidine (5-OH-dC) demonstrated that after 9–24 passages in tissue culture, HIV became undetectable [14]. KP1212, another deoxycytidine analog, typically extinguished HIV in culture by 13 passages [15]. In both studies, an increase in frequency of mutations in viral RNA, predicted by the base-pairing properties of the analog, occurred prior to population collapse. These results are consistent with a phenomenon termed “error catastrophe” in which mutations accumulate with each cycle of infection in the presence of a VDA compound, eventually leading to the loss of the original master consensus sequence and the viral population's collapse [6], [7].

**Figure 1:**
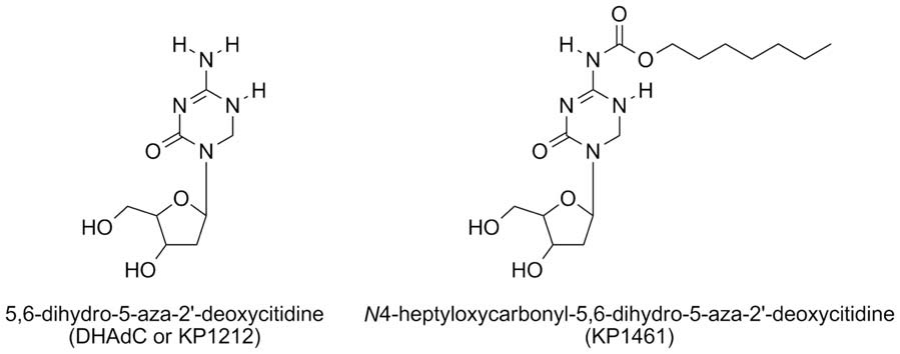
Chemical structures of KP1212 and its prodrug, KP1461, administered in the Phase IIa trial.

KP1212 under its prodrug form – called KP1461 (Figure 1) – was studied in three phase I and one phase II clinical studies. Throughout the remainder of this manuscript, KP1461 will be used to refer to this agent in clinical protocols. In phase I studies, KP1461 was shown to be safe at doses ranging from 100 mg to 3200 mg given twice-daily for a period of 14 days (P. Clay et al, manuscript in preparation).

We now report data that address the proposed mechanism of action of VDA from a secondary analysis of a Phase IIa trial carried out with twice-daily administration of 1600 mg of KP1461 for 124 days. At the end of the clinical study, no sustained reduction in HIV-1 viral load was observed. This was not unexpected as we were unsure to what extent viral populations *in vivo* need to be mutated to achieve a hypothesized collapse, and whether this could have been achieved over the course of a 125-day study (subjects were treated for 124 days and the final sample taken for sequencing on day 125). Hence, we sought to determine if there were evidence for an early, subclinical effect of KP1461, prior to the hypothesized ablation of the viral population. Because results from cell culture experiments suggested that mutations accumulate in viral genomes before a population collapse, we examined the HIV-1 populations derived from the Phase IIa trial for acquisition of excess A to G and G to A mutations in the viral genome consistent with the mechanism of action of the drug [15]. Here we report the direct sequence analysis of over 7.1 million base pairs of unique HIV-1 genomes sequences derived from 105 1.0 kb PCR amplicons from the *gag* gene at each of three time points during therapy in 10 treated subjects and 10 control subjects sampled at approximately the same intervals, as well as from 8 untreated individuals from a Phase I trial of KP1461 therapy. The data revealed an excess of mechanistically anticipated mutations in subjects treated with the drug, suggesting that VDA is applicable to human treatment.

## Results

### Diversity and private mutations

**Figure 2:**
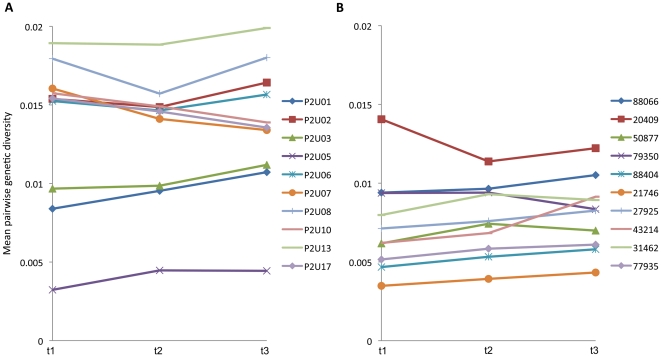
Genetic diversity of 1.0 kb segments of HIV-1 *gag* genes through time in individuals treated and untreated with KP1461. Average population diversity of samplings of 105 1.0 kb *gag* gene segments through time is shown for treated (panel A) and untreated (panel B) groups.

**Figure 3:**
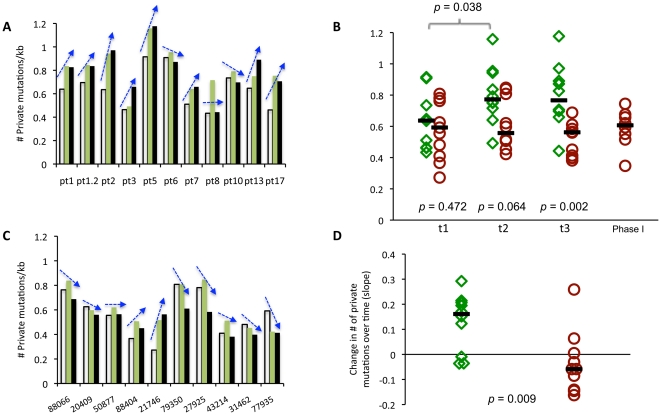
Private mutations detected in HIV-1 *gag* genes in treated and control groups. The average number of private mutations (found in only one of 105 sequences in each sample) per kilobase of sequence are shown for treated (Panel A) and control (Panel C) subjects at three time points and in Phase I trial participants (Clay et al, manuscript in preparation). In panels A and C, open bars are from day 0 (t1), shaded bars are from day 56 (t2) and black bars are from day 125 (t3). Patient 1 underwent 2 courses of treatment, pt1 and pt1.2, with a 9-day gap in between. The trajectory lines drawn on top of each 3-bar data set (for each individual) track the accumulation or loss of mutations over the study. In Panel A, 8 of 11 comparisons revealed an accumulation of mutations, whereas in control participants depicted in Panel C, only 2 0f 10 had an accumulation of mutations over the study. Panel B shows the number of private mutations per subject at each of the three time points. Data from treated subjects are shown with diamond symbols and controls with circles. Phase I trial participants sampled at day 0 (Panel B) are also shown with triangle symbols. Median levels for each group are shown with a horizontal line. Panel D shows the overall change in the number of private mutations over the 125-day sampling period in the treated group and over comparable intervals in the untreated control subjects. Wilcoxon ranked sum tests were used to assess differences in total private site counts within subjects and between treated and untreated groups. In panel B, p values for treated vs. phase I subjects  = 0.894; phase I subjects vs. phase 2 controls  = 0.549.

Viral diversity typically increases during asymptomatic HIV infection and then may decrease thereafter [26]. We did not observe a consistent trend in diversity in these subjects over the 125-day period of examination (Figure 2), perhaps due to the relatively short observation time, to obtaining sequences from a relatively conserved region of the HIV-1 genome, and by sampling individuals in varied disease states. Furthermore, by definition, viral genomes that develop lethal mutations would not reproduce and may be quickly lost from the viral population. Hence, to enhance the sensitivity of our analysis we focused on mutations that reflect the most recent rounds of replication, “private site mutations”, defined as a mutation found in only one of the (N = 105) viral genomes sequenced. As shown in Figure 3, a substantial increase in private site mutations was noted in these subjects (Figure 3A), especially over the first 56 days of treatment (Figure 3B, p = 0.038).

To place these observations into context we performed a parallel analysis of 10 untreated subjects. Based on accepted ART treatment guidelines, clinicians and institutional ethics boards were not supportive of including a placebo group where ART-experienced subjects would be kept off any treatment regimen for the study duration of 4+ months. While no similarly advanced, ART-experienced subjects were available with the short (∼2 m) sampling intervals evaluated in the Phase IIa trial, we were able to closely match the sampling intervals in a cohort of longitudinally studied volunteers initially enrolled in primary HIV infection [27], and infected for an average of 3 years and ART naïve when evaluated here (Table 1). As expected from their shorter period of infection, the controls typically had a lower overall level of quasispecies diversity than treated individuals (Figure 2B), and like the treated individuals, exhibited no large change in diversity over the 4-month study. In contrast to treated subjects, however, no significant change in the numbers of private mutations was observed between any of the three time points (Figure 3B and C). A second comparison group for day 0 mutation levels was derived from a Phase I trial of KP1461 (Clay et al, manuscript in preparation) (Figure 3B, rightmost column), who, like the Phase IIa trial participants, had more advanced infection. No significant difference in the numbers of private mutations was observed between the Phase I subjects and either of the other two groups at day 0 (Figure 3B). The Phase 1 study was carried out for only two weeks and there was no significant increase in private mutations. Treated, control and Phase I groups had similar numbers of private mutations at Day 0 (Figure 3B, p = 0.427 for treated vs. controls and 0.894 for treated vs. Phase I subjects), but differences between the treated and controls groups in the Phase IIa trial increased over time (p = 0.064 at day 56 and p = 0.002 at day 125, Figure 3B). As shown for treated participants in Figure 3A, 8 of 11 comparisons revealed an accumulation of mutations, whereas in control participants depicted in Figure 3C, only 2 of 10 had an accumulation of mutations over the study period. Overall, there was a significantly greater increase in private site mutations in the treated group compared to the control group over the entire period of analysis (Figure 3D, p = 0.009).

**Table 1:** Demographics of treated and control groups.

	Treated	Controls	Phase 1 Subjects
**# Subjects**	10	10	8
**Prior ART**	Yes	No	Yes
**Median years since HIV diagnosis (range)** *	15.5 (<1–23)	2.5 (2.1–6.2)	13.0 (8–21)
**Log median viral load (range)** *	5.23 (4.17–5.88)	4.09 (3.50–5.03)	4.76 (4.15–5.23)
**Median CD4 cells (range)** *	293 (133–485)	387 (251–769)	261 (122–710)
**Sampling times in days**	0, 56, 124	0, 49–78, 105–147	0, 14

*At Day 0 of treatment.

Interestingly, one subject (pt.1) underwent two cycles of 124 day KP1461 treatment following a 9-day interruption. Prior to the second cycle of treatment, at the end of this interruption, the number of private mutations decreased to near baseline levels and then increased during the 2^nd^ cycle of treatment to the levels observed during the initial course of treatment (Figure 3A, pt1.2). This result suggests that mutated viruses are quickly lost upon cessation of KP1461 therapy, and that new mutants are generated upon reapplication of the drug.

### Mutational changes characterizing the treated and control group populations

**Figure 4:**
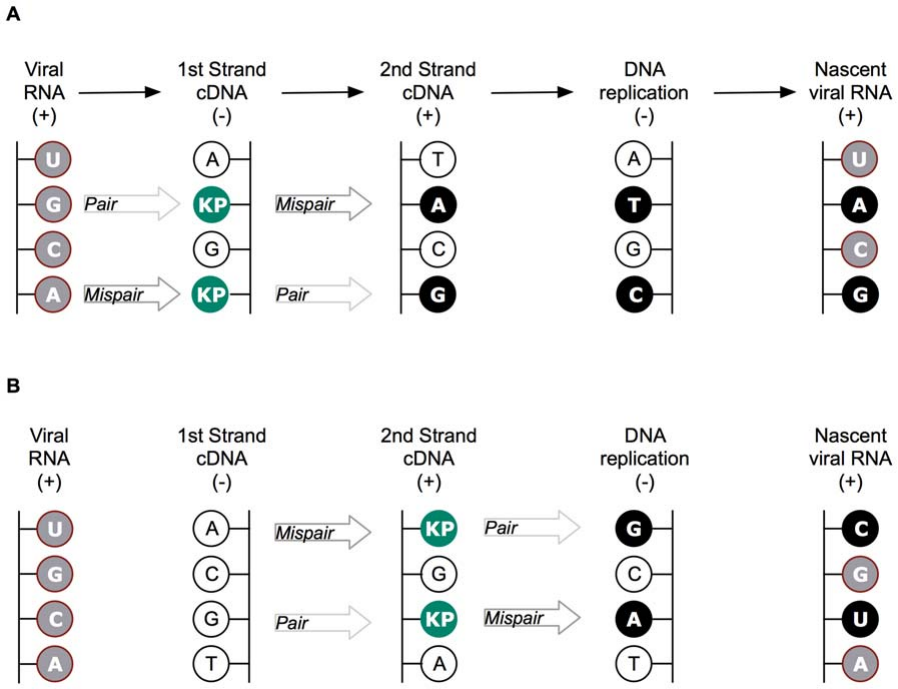
KP1461 mediated mutagenesis due to promiscuous base pairing. **A**. KP1461 incorporation into the 1st cDNA strand, with or without proviral DNA repair. As a C-analogue, KP1461 is expected to pair with G, we refer to pairing with A as a mispair. The KP nucleotide is shown in green circles, whereas natural nucleotides that are incorporated as a result of KP1461 mispairing are in black. Unaffected DNA nucleotides are in white circles and RNA nucleotides are in grey circles. **B**. KP1461 incorporation into the 2nd cDNA strand, no repair of proviral DNA. No mutations would result from incorporation of KP1461 in the second cDNA strand if repair/removal of the KP1461 base following provirus formation.

The types of private site mutational changes in treated and control individuals were examined to discern whether excess drug-specific mutations had emerged in participants who had experienced KP1461. Given that the drug is a deoxycytidine analog that can base pair with A as well as G [15], we pre-specified the hypothesis that there would be an increase in G to A and A to G single-based paired substitution mutations, and to a lesser extent T to C and C to T mutations (Figure 4), as the T/C mutations require persistence of KP1461 in proviral DNA and could be removed by nuclear DNA repair mechanisms. KP1212 is not likely to be excised when present in the DNA strand of an RNA:DNA hybrid but is subject to removal by glycosylases when present in double-stranded DNA [28]. In other studies, we have shown that KP1212 undergoes the predicted tautomerizations to allow base pairing with both G and A (D. Li et al, manuscript in preparation), and that KP1212 triphosphate is incorporated by HIV reverse transcriptase, and when present in an RNA template, directs synthesis of DNA by HIV reverse transcriptase *in vitro* [29] {Murakami, 2005 #1493}(Shen, Venkanatsan and Loeb, et al, unpublished).

**Figure 5:**
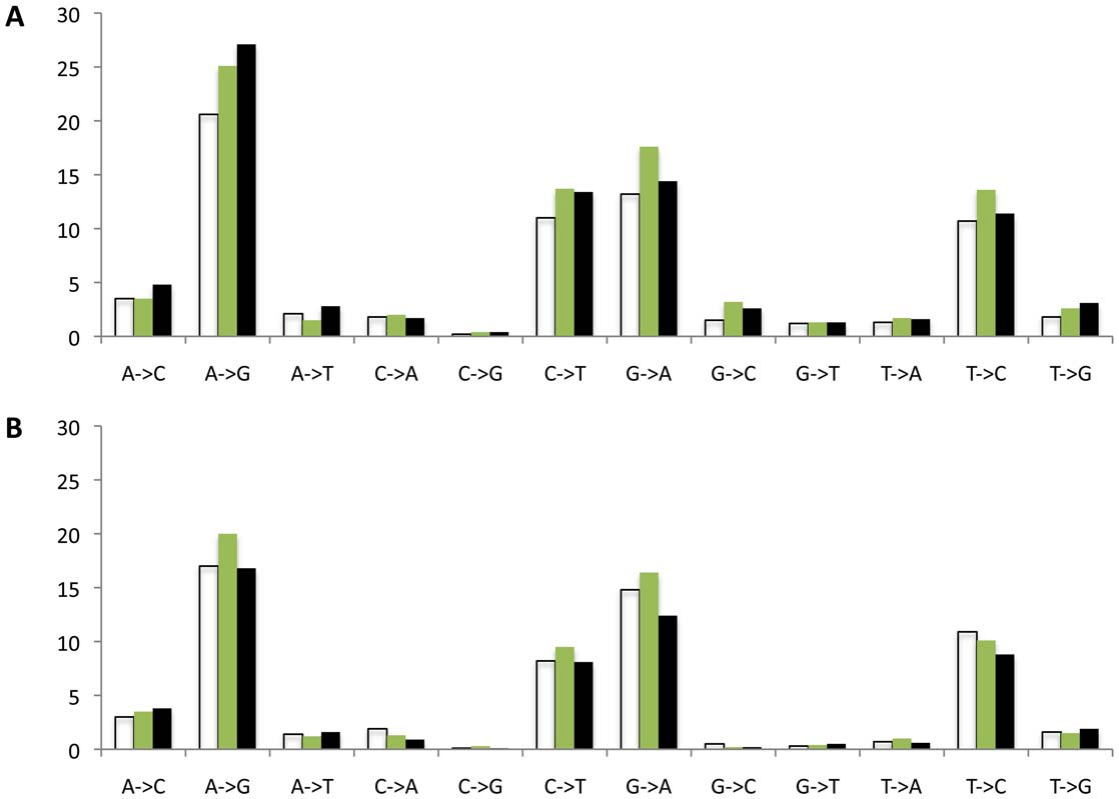
Specificity of private mutational change over time in the treated (panel A) and control (panel B) groups. Open bars are from day 0, shaded bars are from day 56 and black bars are from day 125. The y-axis shows the total number of private mutations in the two groups of 10 subjects.

As shown in Figure 5, the frequency of A to G mutations was higher in treated participants (mean  = 20.6, Figure 5A) compared to controls (mean  = 17.0, Figure 5B) at baseline (mean difference  = 3.6; p = 0.29). After subtracting this baseline difference, the treated participants had 1.5 and 6.7 more A to G mutations at days 56 and 125 days, respectively, relative to the controls (Table 2). In addition, they had 2.8 and 3.6 more G to A mutations than the controls at days 56 and 125, respectively. Overall, treated subjects had significantly more A to G and G to A mutations (p = 0.01) as well as all transitions (p = 0.01) than the control group (there was a trend toward increased T to C plus C to T mutations (p = 0.09)). Taken together, and correcting for multiple testing, all transition mutations in the treated group were substantially increased over controls (p = 0.03). Pre-specified analyses thus reflected the expected effect of KP1461**.** Exploratory analyses of the other mutations were conducted to understand the full spectrum of change during treatment: There was no statistically significant increase in any of the other 8 possible mutation types, including all transversions, and, the overall p-value for any effect of treatment on transversion mutations, corrected for multiple testing, was 0.38. Hence, these mutations were not statistically enhanced.

**Table 2:** Differences in private site mutations observed in treated and control groups.

	Day 0	From day 0 to day 56		From day 56 to day 124		From day 0 to day 124	
	Mean^1^	Mean difference^2^	p-value^3^	Mean difference^2^	p-value^3^	Mean difference^2^	p-value^4^
Predefined hypotheses							
AG	18.8	1.5	0.53	5.2	0.14	6.7	0.10
GA	14.0	2.8	0.10	0.8	0.76	3.6	0.29
AG+GA	32.8	4.3	0.12	6.0	0.05	10.3	0.01
TC	10.8	3.7	0.10	−0.9	0.47	2.8	0.14
CT	9.6	1.4	0.47	1.1	0.65	2.5	0.28
CT+TC	20.4	5.1	0.10	0.2	0.92	5.2	0.09
All Transitions	53.2	9.4	0.01	6.2	0.10	15.6	0.01
P-value for testing for a treatment effect on any transition or combination of transitions at any time, correcting for multiple testing: 0.03							
Exploratory hypotheses							
AC	3.25	−0.5	0.58	1.0	0.40	0.5	0.64
CA	1.85	0.8	0.49	0.1	0.88	0.9	0.31
AT	1.75	−0.4	0.56	0.9	0.46	0.5	0.55
TG	1.7	0.9	0.11	0.1	0.83	1.0	0.36
TA	1.0	0.1	0.84	0.3	0.64	0.4	0.50
GC	1.0	2	0.03	−0.6	0.60	1.4	0.04
GT	0.75	0	0.90	−0.1	0.99	−0.1	0.99
CG	0.15	0	0.82	0.2	0.71	0.2	0.71
All Transversions	11.4	2.9	0.26	1.9	0.55	4.8	0.09
P-value for testing for a treatment effect on all exploratory hypotheses, correcting for multiple testing: 0.38							

1 Mean number of private site mutations over all participants at time 0.

2 Difference (treated minus control) after subtracting the difference at baseline.

3 All p-values are two-sided and based on permutations tests with 1000 repetitions.

4 Difference (treated minus control) at day 124 minus difference at day 56.

## Discussion

In light of increasing drug resistance to currently available treatment regimens and the continuing search for an effective HIV-1 vaccine, novel therapeutic interventions are warranted. Lethal mutagenesis, or VDA, has emerged as a potential therapy option in recent years, by targeting HIV-1's strongest asset, its quasispecies nature and ability to diversify beyond immune and drug inhibition in short time intervals. It is now appreciated that lethal mutagenesis is a strategy our innate immune system may also use to fight HIV. Members of the APOBEC family of proteins encode an activity that can potently deaminate cytosines in nascent HIV cDNA, leading to G to A mutations, as well as G to A hypermutation in the absence of a functional HIV viral infectivity factor (Vif) protein (see [30] for a recent review). The present study is the first to examine the genetic effects *in vivo* of a novel, externally applied mutagen of HIV-1. Thus, the APOBEC enzymes may function synergistically with KP1461 to enhance HIV mutagenesis. In serial passage experiments in the presence of a VDA agent, viral cultures maintain their titers until sufficient mutational burden has accrued to result in population collapse [14]–[25]. Because we did not see a significant decrease in viral load in the treated subjects (though a trend toward a 0.32 log decrease was observed) (Hicks et al, manuscript in preparation), it is likely that the treatment time was not long or intensive enough to achieve population collapse. Improvement in formulation and a more sustained treatment regimen may allow sufficient mutations to accumulate. The increase in the number of VDA-anticipated mutations in the viral genomes of treated participants is an important sign of therapeutic efficacy and establishes that nucleotide analogs can increase the frequency of mutations in the HIV genome *in vivo*. In addition, KP1461 exerts its influence without immediately interfering with viral replication (such as occurs with chain terminator and protease inhibitor drugs), which in turn would be expected to delay development of resistance [31].

Because HIV-1's mutation rate is close to the error threshold, the increase in the mutational frequency needed to collapse the population may be small. For example, HIV-1 mutation frequencies in the envelope gene increase by 40-90% (including private and non-private changes) in cell culture following exposure to KP1212 [15]. We observed an increase in private mutation frequencies that averaged 20% above that of controls, with little additional increases noted after 2 months of therapy (Figure 3). Interestingly, the one subject that underwent a second cycle of therapy after an 9-day interruption, rebounded to approximately the same 20% increase in private mutations that was observed during his first course of therapy.

It is not possible to simply extrapolate from previous cell culture mutation data to the *in vivo* tests in order to predict if or how long it may take to bring the *in vivo* population to the point of collapse. This is due to the presence of viral reservoirs, compartments, immune clearance, a greater variation of drug dosage and perhaps a differential impact of recombination *in vivo*, and a likely slower clearance rate of virions in cell culture. *In vivo* then, we are faced with the task of searching for viruses that are eliminated quickly in the body. Hence, our experimental plan was to examine mutations occurring as close to the most recent round of viral replication as we can get, prior to propagation in the viral population – private sites represent this class of mutations. Previous cell culture studies also used a clonal laboratory strain of HIV (NL4-3) and sequencing was carried out after cloning [14], [15], not a quasispecies examined by single genome sequencing as was the case for the present study. The impact of these differences may be substantial – we cannot evaluate private sites adequately in molecularly cloned sequences as the cloning process fixes PCR errors; while such errors are not observed when examining direct sequencing data from single amplified viral templates as done here. Conversely, we can examine the total evolution of the viral population from the starting inoculum in cell culture, but cannot in most cases do this *in vivo*.

Observable levels of private mutations are likely to be deceptively low, especially *in vivo*, as two mitigating processes occur: viruses that accrue substantial mutational burdens are defective and are lost from the population, and recombination can act to preserve the population for at least a time by recombining non-lethally mutated regions of viral genomes. Our focus on the measurement of private mutations brings us as close as possible to observing single-cycle mutation rates *in vivo*, as by definition, these mutations are only found in single viruses within our sampling. However, our extensive and accurate sampling (105 single genome templates per time point), while being unprecedented for Sanger sequencing and matching the sensitivity of current massively parallel sequencing technologies [32], still only samples a small fraction of the viruses produced in a person each day and thus overestimates private mutation frequencies.

The longitudinally followed treated and control groups were infected for disparate time periods (Median 15.5 years in the treated and 2.5 years in the controls). However, that the treated, control and Phase I groups (the latter group was infected for a median of 13 years) had very similar levels of private mutations at day 0 argues that the increased levels of mutations we observed in the treated populations over time were not due to higher overall levels of private mutations in later stages of infection. The slightly higher baseline levels of private mutations observed in the individuals with longer periods of infection were small and non-significant compared to the increases noted upon receiving KP1461.

KP1461 treatment resulted in a significant increase in transition mutations, especially A to G and G to A. Because such transitions occur frequently in natural HIV infections, it is highly unlikely that KP1461 will create new resistant viruses not normally observed in nature or induced by APOBEC interactions. Moreover, since the preponderance of mutations will be deleterious [8], this therapeutic strategy may diminish the vitality of the viral population. In the treated participants, G to C mutations were also increased, although the overall number of these mutations was small and the increase was not seen after day 56. Such an increase in G to C mutations recently found a possible explanation in the work of Mansky and colleagues [17], who showed that another C-analogue with the capacity to increase HIV mutation rates and extinguish HIV populations in cell culture, 5-azacytidine (5-AZC), could induce G to C transversion mutations in HIV-1.

Subjects participating in the Phase IIa trial were extensively ART-experienced and chronically infected with HIV-1 for a median of 15 years. While these subjects likely entered the trial with pre-existing antiretroviral resistance already selected for, KP1461 did not enhance the levels of resistance mutations, or select for new resistance mutations to known therapies. Resistance to VDA drugs such as KP1461, should it occur, may take the form of increased replication fidelity, which should result in a decrease in overall diversity and lead to propagation of less fit virus that is also less able to evolve resistance against standard antiretrovirals [25], [33]. Because of that potential and because KP1461 is safe and well tolerated, it is conceivable that KP1461 would increase the efficiency of current inhibitors (e.g., NRTI, NNRTI, protease inhibitors), and potentially be useful as a sequential monotherapy [34]. VDA drugs such as KP1461 might also find use in failing ART regimens in which drug-resistance results in a partial loss in replication fitness [35], as such viruses may be even closer to the hypothesized brink of population collapse than drug sensitive viruses.

The results reported here derive from the first time a VDA agent has been used to treat HIV infections *in vivo*. The regimen employed imparted no clinical benefit and there are many aspects of the pharmacodynamics and pharmacokinetics that still need to be studied. Different formulations and treatment regimens may improve clinical results. Further studies are needed to examine the full potential impact of KP1461 *in vivo*, either as a monotherapy or as an addition to both suppressive and failing ART regimens. Importantly and unlike traditional antiretrovirals, viral decay accelerators are also predicted to have an impact on the population of latently infected cells, since latent HIV-1 infected cells are activated at low levels and virus reservoirs can be reseeded [36]–[38], albeit very slowly or possibly not appreciably in strongly adherent subjects [39]. Contrary to traditional antiretroviral drugs, we would expect VDA drugs to have an effect on active reservoirs because, as the reservoirs are reseeded, they would become increasingly populated with excessively mutagenized viruses with potentially ratcheted-down fitness. This condition (referred to as Muller's ratchet) is only applicable to small populations [40], [41], which can be met in the case of HIV-1 under effective therapy, as the replicating pool of virus becomes very small and there are increasingly fewer viruses available for the restoration of viability due to recombination. In light of frequent toxicities resulting from existing antiretroviral treatments, along with the emergence and transmission of resistant variants, VDA drugs such as KP1461 could offer a new and valuable addition in the arsenal of tools for the treatment of HIV-1. Furthermore, similar therapeutic agents can be brought to bear on other important and genetically variable human pathogens for which there are even fewer therapeutic options, such as hepatitis viruses.

## Materials and Methods

### Ethics Statement

10 adult subjects were recruited through Clinical Trial NCT00504452 and 10 more from the University of Washington Primary Infection Clinic (PIC), and gave written informed consent under clinical protocols approved by the University of Washington Institutional Review Board.

### Specimens

Details of the Phase IIa clinical trial of KP1461 (ClinicalTrials.gov identifier NCT00504452) will be provided elsewhere (C. Hicks et al., manuscript in preparation). In brief, it was an open-label, single-arm, multicenter, mechanism validation study of KP1461 as monotherapy in antiretroviral-experienced HIV subjects. Subjects did not take standard ART for at least 4 months prior to the start of the trial. No placebo control group was included, as based on accepted ART treatment guidelines, clinicians and institutional ethics boards were not supportive of including a placebo group where ART-experienced subjects would be kept off any treatment regimen for 8+ months. A 1600 mg dose of KP1461 was administered twice daily for 124 days to a total of 24 participants, 13 of whom completed the treatment. No consistent dimunution in viral load was observed during the trial. Treatment with KP-1461 was, in general, well tolerated. The most frequently reported adverse events were mild to moderate self-limiting conditions, such as upper respiratory tract infection, sinusitis, headache, and pharyngolaryngeal pain. No treatment-emergent serious adverse events were reported that were attributed to study drug. Genotype and phenotype results indicated that exposure to KP-1461 did not result in increased resistance to approved antiretroviral drugs. Frozen blood plasma specimens were obtained from the first 10 phase IIa trial participants to complete KP1461 dosing in the trial at day 0, day 56 and one day after the conclusion of treatment trial on day 125. To assess viral evolution in untreated subjects over similar periods of time, blood plasma from untreated HIV-1 infected control subjects were derived from the Seattle Primary Infection Clinic Cohort [27] (Table 1). Similarly, baseline measurements of mutations from a Phase I trial of KP1461 (P. Clay et al, manuscript in preparation) were included as a separate comparison group.

### RNA extraction and cDNA synthesis

Extraction of viral RNA from blood plasma was performed using the commercially available QIAamp Viral RNA Mini Kit (Spin Protocol). cDNA synthesis was conducted in a 20 uL reaction containing RNA (11.5 uL), 10 mM dNTP, 5x Reaction Buffer, 0.1M DTT, SuperScript III reverse transcriptase, RNase Out and either 50 uM Oligo-dT or 5′-GCCTTATGGCCGGGTCCTCCCACTC-3′ (HXB2 positions 1868-1844) as primers. RNA, dNTPs, and Oligo-dT were mixed and incubated at 65°C for 5 minutes and then placed on ice. 5x Superscript Reaction Buffer, 1M DTT, SuperScript III, and RNase Out were incubated separately at 45°C for 5 minutes. The two solutions were then mixed and incubated at 45°C for 90 minutes, followed by 70°C for 15 minutes. RNase H was added and incubated at 36°C for an additional 20 minutes.

### Single-template PCR amplification

All of the sequence data reported here were obtained using our established procedures for the PCR amplification and direct sequencing of PCR products derived from individual HIV-1 templates [3], [42]–[44]. Direct sequencing, producing a consensus of sequences derived from a single viral template, was used to avoid detection of mutations introduced during PCR. Briefly, end-point dilution of cDNA followed by nested PCR was performed in order to quantify the number of amplifiable copies of viral template per volume of cDNA. An initial quantification plate was prepared for each sample starting with an initial 1∶10 dilution of cDNA followed by nine 1∶1 serial dilutions in triplicate. The copy number per volume of cDNA was calculated using the Quality tool [43] (http://indra.mullins.microbiol.washington.edu/quality/) after scoring for 2nd round PCR products on a 1% agarose gel. Following quantification, nested PCR was conducted in 96 well plates using a dilution that deposited approximately one amplifiable template per 5 wells. A total of 144 PCR products were obtained per sample.

First round PCR consisted of 1.0 uL of cDNA in a 25 uL reaction containing 1x Bioline NH_4_ PCR buffer, 1.7 mM MgCL_2_, 200 uM of each dNTP, 2 units of Biolase Taq Polymerase, and primers 5′-TGACTAGCGGAGGCTAGAAGGAGAGAG-3′ (HXB2 positions 763–789) and 5′-GCCTTATGGCCGGGTCCTCCCACTC-3′ (1868-1844). Round two consisted of 2 µL of Round 1 product in a 25 µL reaction as above, and primers 5′-GTGCGAGAGCGTCGGTATTAAG (2C/1T)G-3′ (794-817) and 5′-ACTCCCTGACATGCTGTCATCA-3′ (1847-1826). Temperature cycling conditions for both rounds of PCR were: 95°C for 2 minutes, 30 cycles (95°C 15 seconds, 60°C 30 seconds, 72°C 1 minute), 72°C for 7 minutes, and 4°C to hold.

### DNA Sequencing

PCR products were sequenced using the Sanger method. Sequence chromatograms were checked for accuracy and edited in Sequencher 4.7 (Gene Codes Corp., Ann Arbor, MI, USA) –sequences with ambiguous base calls due to multiple peaks were excluded from further analysis. Sequences were trimmed to include HXB2 *gag* nucleotide position 27 to 1043 (816 to 1832 from start of the HXB2 genome), and sequences from each subject were aligned using MUSCLE v3.7 [45]. Alignments were manually refined in MacClade v4.08 (Sinauer Associates, Inc., Sunderland, MA). All sequences were checked for sample mixup or contamination via Viroblast [46] (http://indra.mullins.microbiol.washington.edu/viroblast/viroblast.php) searches against local and published sequence databases and by observing that sequences from each subject clustered separately from every other subject in Jukes-Cantor phylogenetic trees calculated in Seaview [47] from an alignment of all sequences from all subjects. One hundred and five high-quality sequences from each time point were included for all further analyses. All of the sequence data (8,285 *gag* gene sequences) has been deposited in GenBank (Accession numbers HQ248229-HQ256513).

Viral diversity for each time point was calculated in DIVEIN [48] as pairwise distances under the maximum likelihood model GTR+G+I. Statistical comparisons of diversity between time points were performed using the two-sample tests for comparing intra-individual sequence diversity between populations [49] (http://www.scharp.org/users/adecamp/diverstest/runtests.php); comparisons within each individual were calculated using the Tpoolmedian test, which accounts for multiple comparisons inherent in a pairwise diversity matrix, while the comparisons between time points among all of the subjects pooled were performed with the Tsubjmean test, which treats the averages of the pairwise distances within each individual as the observations.

Private mutations in each time point in each subject were calculated using the InSites function within DIVEIN [48], in which a private site is defined as a site where only one sequence in the complete alignment has a base that is different from the consensus state. InSites calculates total private sites found in an alignment, and also counts the types of changes observed (i.e., if at a given site, the consensus state is A and one sequence has a G at that site, the tally is increased for “A to G” type private mutations). Comparisons of total private site counts, between time points within subjects, and between the treated and untreated groups at each time point, were done using the Wilcoxon Rank Sums test. The slope of the line made by the private site count through all three data points was calculated for each subject and plotted by treatment group; comparison of slopes between treatment groups was done using the Wilcoxon Rank Sums test.

### Statistical analyses of the drug effect on number of mutations

We tested for differences in various types of mutations between cases and controls at days 56 and 125, after subtracting the difference at time 0. The analysis is based on the following mixed effects {Laird, 1982 #3869} model
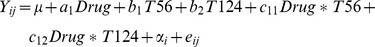
where Y_ij_ is the outcome (number of mutations) for person i at time j, a, b, and c are parameters that capture the drug effect, time effect and interactions, respectively, α_i_ is a random person effect (α_i_ ∼ N(0,τ^2^)) and e_ij_ is a random error (e_ij_ ∼ N(0,σ^2^)). These parameters are estimated from the data using restricted maximum likelihood methods {Laird, 1982 #3869}. Thus, the model accounts for correlation between the repeated observations over time in an individual. To ensure robustness with respect to the model assumptions regarding normality, p-values to test the change in mutations associated with treatment (i.e., treatment minus control) from time 0 to time 56 (c_11_), from time 0 to time 126 (c_12_) and from time 56 to time 125 (c_12_ – c_11_) were computed using permutation tests {Edgington, 1995 #3868} with 1000 replications. A permutation procedure was also used to compute two p-values that account for multiple testing. The first permutation procedure was used to compute the null distribution of the maximum treatment effect for the pre-specified effect of KP1461 on any transition (A to G, G to A, AG+GA, C to T, T to C, CT+TC and/or all transitions) over the 3 possible time intervals (0 to 56, 0 to 125 and 56 to 125). This p-value provides a type I error rate for any significant finding in the 21 hypothesis tests used to address the question “Is there a treatment effect on transitions at any time point?” The second permutation procedure was used to compute the null distribution of the maximum treatment effect for all other hypothesis tests (all other individual mutations and all transversions). The p-value from this test addresses the question “Is there a treatment effect on any other type of mutation at any time point?”
